# The complete plastid genome of *Abrus pulchellus* subsp. *mollis* (Leguminosae): a medicinal plant in Southern China

**DOI:** 10.1080/23802359.2024.2383684

**Published:** 2024-07-29

**Authors:** Cai-Yun Zhang, Wei-Fen Qiu, Yu-Zhen Chen, Xiao-Lu Mo, Hai-Fei Yan

**Affiliations:** a Guangdong Food and Drug Vocational College, Guangzhou, China; b Key Laboratory of Plant Resources Conservation and Sustainable Utilization, South China Botanical Garden, Chinese Academy of Sciences, Guangzhou, China

**Keywords:** *Abri Herba*, Leguminosae, chloroplast genome, genome skimming, phylogenetic analysis

## Abstract

The subspecies *Abrus pulchellus* subsp*. mollis* exhibits pharmacological properties akin to the traditional Chinese medicinal plant *Abri Herba* (*A. pulchellus* subsp*. cantoniensis* (Hance) Verdc.). In this report, we unveil the plastid genome of *A. pulchellus* subsp*. mollis*. The genome spans 156,322 base pairs (bp), comprising a large single-copy (LSC) region of 86,633 bp, a small single-copy (SSC) region of 18,219 bp, and two distinct inverted repeat regions (IRs) of 25,735 bp each. Annotation process cataloged a total of 111 genes within this genome, including 77 protein-coding genes, 30 transfer RNA (tRNA) genes, and four ribosomal RNA (rRNA) genes. The overall guanine-cytosine (GC) content of the plastome is 35.5%. Phylogenetic analysis utilizing maximum-likelihood (ML) based on 16 complete plastid genomes reveals a close clustering of three *Abrus* taxa, namely *A. pulchellus* subsp*. mollis*, *A. pulchellus* subsp*. cantoniensis*, and *A. precatorius*. Notably, *A. pulchellus* subsp*. cantoniensis* clusters with *A. precatorius* as a sister group, distinct from *A. pulchellus* subsp*. mollis*. These findings highlight significant differences between the plastid genomes of the two subspecies, laying the foundation for future research on the identification of medicinal herbs and germplasm resources related to these subspecies.

## Introduction

The *Abrus* Adans. (Leguminosae) encompasses approximately 17 species globally, distributed across the tropical and subtropical region of the old world to the Southwest Pacific (https://powo.science.kew.org). In China, two species, *Abrus precatorius* L. and *A. pulchellus* Wallich ex Thwaites are documented within this genus (Bao and Gilbert 2010). Additionally, *A. pulchellus* is differentiated into three subspecies (Bao and Gilbert 2010). The subspecies *A. pulchellus* subsp. *cantoniensis* (Hance) Verdc., colloquially known as *Abri Herba* or ‘Jigucao’ in Chinese (Chinese Pharmacopoeia Commission 2020), is widely utilized in traditional Chinese medicine for treating hepatitis and liver fibrosis and as a component in herbal teas and soups in southern China (Shen et al. 2021). Conversely, *Abrus pulchellus* subsp. *mollis* (Hance) Verdc. 1871 shares pharmaceutical properties and is commonly used as *Abri Herba* in practical applications. Morphologically, these two subspecies are indistinguishable. This study presents the plastid genome of *A. pulchellus* subsp. *mollis* to facilitate future authentication and quality control of the herbal medicine.

## Materials and methods

**Figure 1.**
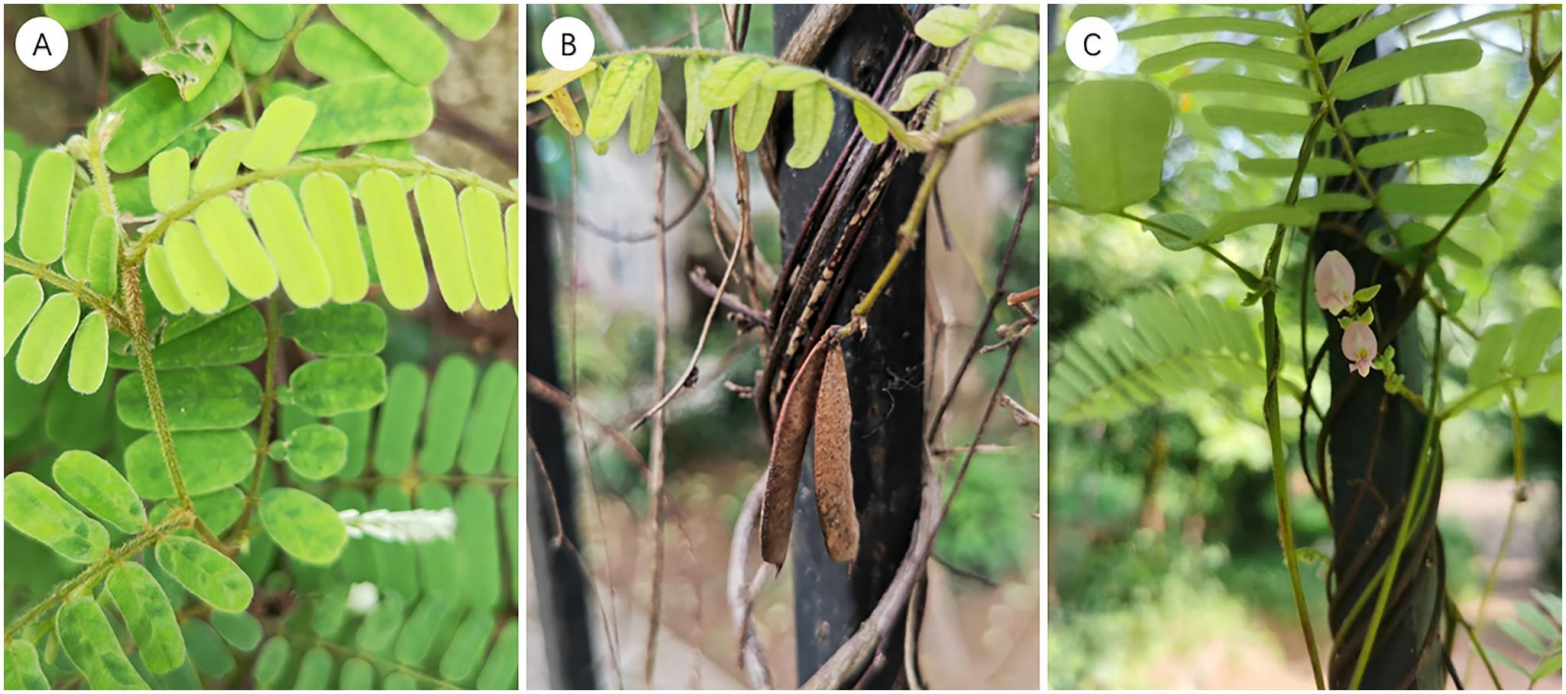
The species reference images of *Abrus pulchellus* subsp. *mollis*. The photographs were taken by Puyue Ouyang at Guangdong Food and Drug Vocational College. The species’ indumentum is at least partially ferruginous, predominantly erect, and often gray on leaves. If the indumentum is gray, the leaflets are approximately 3 cm in length, and the pod is 3.5–4.9 cm in length.

The specimen of *A. pulchellus* subsp. *mollis* was collected from Guangdong Food and Drug Vocational College, Guangzhou, China (23°12′27″N, 113°22′15″E). The voucher specimens (voucher accession: Yan2021187; specimen number: 840735), identified by Cai-Yun Zhang, is archived in the herbarium of South China Botanical Garden, CAS (Fei-Yan Zeng, zengfeiy@scbg.ac.cn) (Figure 1). Genomic DNA was extracted from silica-gel dried leaves using a modified CTAB method (Doyle and Doyle 1987), and a paired-end (PE) library (2 × 150 bp) was sequenced on Illumina HiSeq 2000 at Azenta US, Inc. (Azenta, Suzhou, China).

A total of 7,313,705 raw reads were evaluated using FastQC 0.11.5 (http://www.bioinformatics.babraham.ac.uk/projects/fastqc/), and adapters and low-quality nucleotide bases were trimmed using Trimmomatic 0.35 (Bolger et al. 2014). Subsequently, approximately 2 Gb of clean data was assembled into a plastome using GetOrganelle v1.7.5 (Jin et al. 2020), with visualization and refinement conducted via Bandage v0.8.1 (Wick et al. 2015). The resulting plastome was manually checked by mapping the clean PE reads to the genome using Bowtie2 v2.4.5 (Langmead and Salzberg 2012).

The plastome was annotated using GeSeq (Tillich et al. 2017) and checked with two existing *Abrus* plastomes (MN709888 and MT328396) in GenBank, and tRNA genes were identified using ARAGORN (Laslett and Canback 2004). The complete plastid genome sequence has been deposited in GenBank (accession no. LC708259). The structure of the cis-/trans-splicing genes was generated by CPGView (Liu et al. 2023). Protein-coding genes (CDS) extracted from three *Abrus* plastomes and 13 species from seven closely related genera within Millettioid/Phaseoloid clade of Leguminosae were aligned using MAFFT v7.490 (Katoh and Standley 2013), and used for the maximum-likelihood (ML) tree construction with RAxML v8.2.12 (Stamatakis 2014) with 200 rapid bootstrap replicates.

## Results

**Figure 2.**
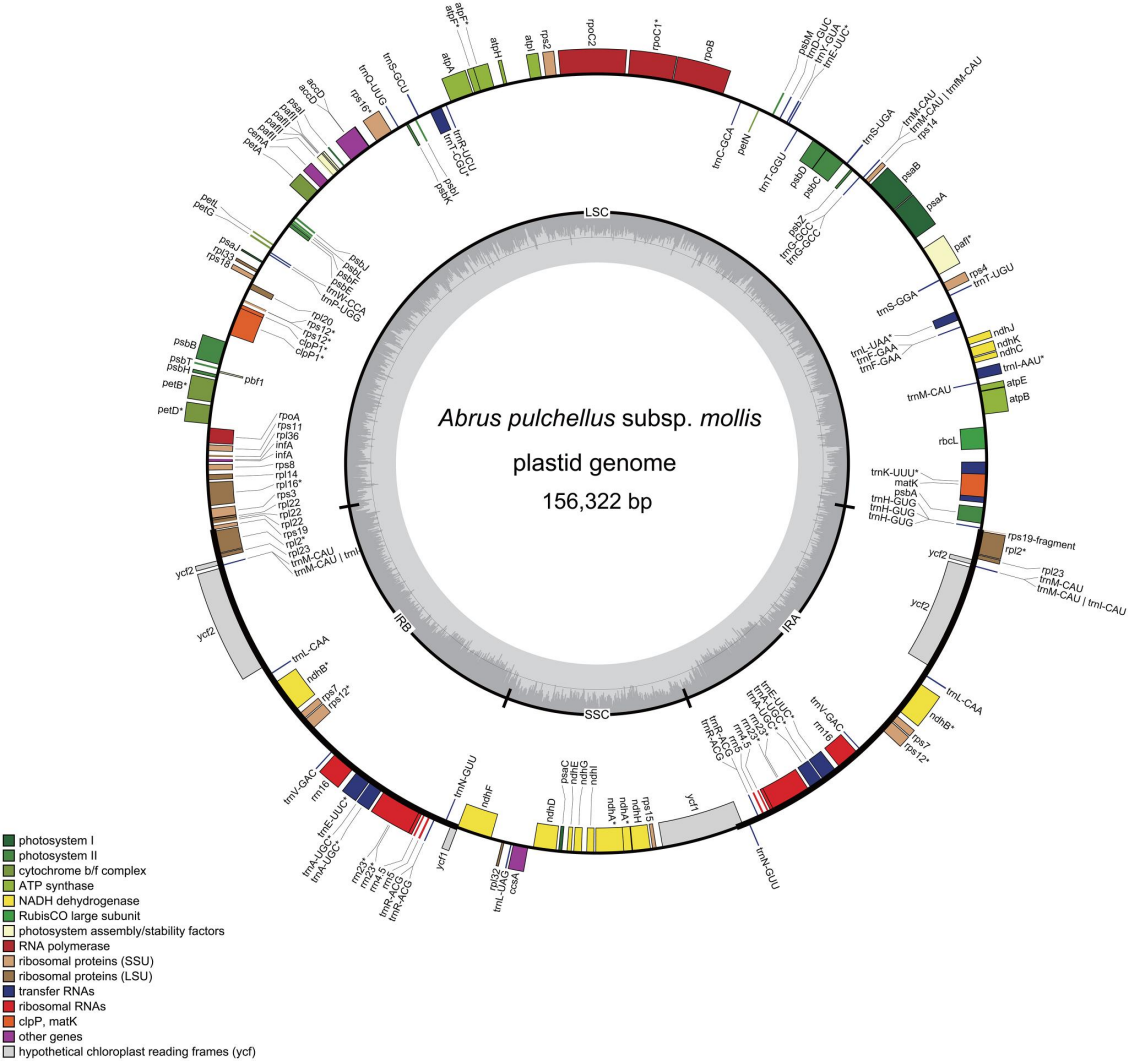
Gene map of the plastid genome of *Abrus pulchellus* subsp. *mollis*. Genes outside the circle are transcribed counterclockwise, and genes inside the circle are transcribed clockwise. The gray graph in the inner circle shows the GC content with 50% threshold line.

The high coverage depth (the average coverage was 1427×, Figure S1) of the resulting plastome of *A. pulchellus* subsp. *mollis* demonstrates the accuracy of the plastome we assembled. The plastome measures 156,322 base pairs (bp), featuring a large single-copy (LSC) region of 86,633 bp, a small single-copy (SSC) region of 18,219 bp, and two distinct inverted repeat regions (IRs) of 25,735 bp each (Figure 2). In total, 111 genes were annotated, including 77 CDS, 30 transfer RNA (tRNA) genes, and four ribosomal RNA (rRNA) genes. Notably, 17 genes are duplicated due to their location within the IR regions. Fifteen genes, such as *rpoC1*, *atpF*, *rps16*, harbor a single intron, while *clpP* possesses two introns. The *rps12* is identified as a trans-splicing gene, comprising three exons and one intron, with one exon situated in LSC region, separate from the remaining segments in the IR regions. The gene structure maps of cis- and trans-splicing gene are shown in Figures S2 and S3. The overall guanine-cytosine (GC) content of the plastome stands at 35.5%, with the LSC, SSC, and IR regions having GC contents of 32.8, 28.8, and 42.4%, respectively.

**Figure 3.**
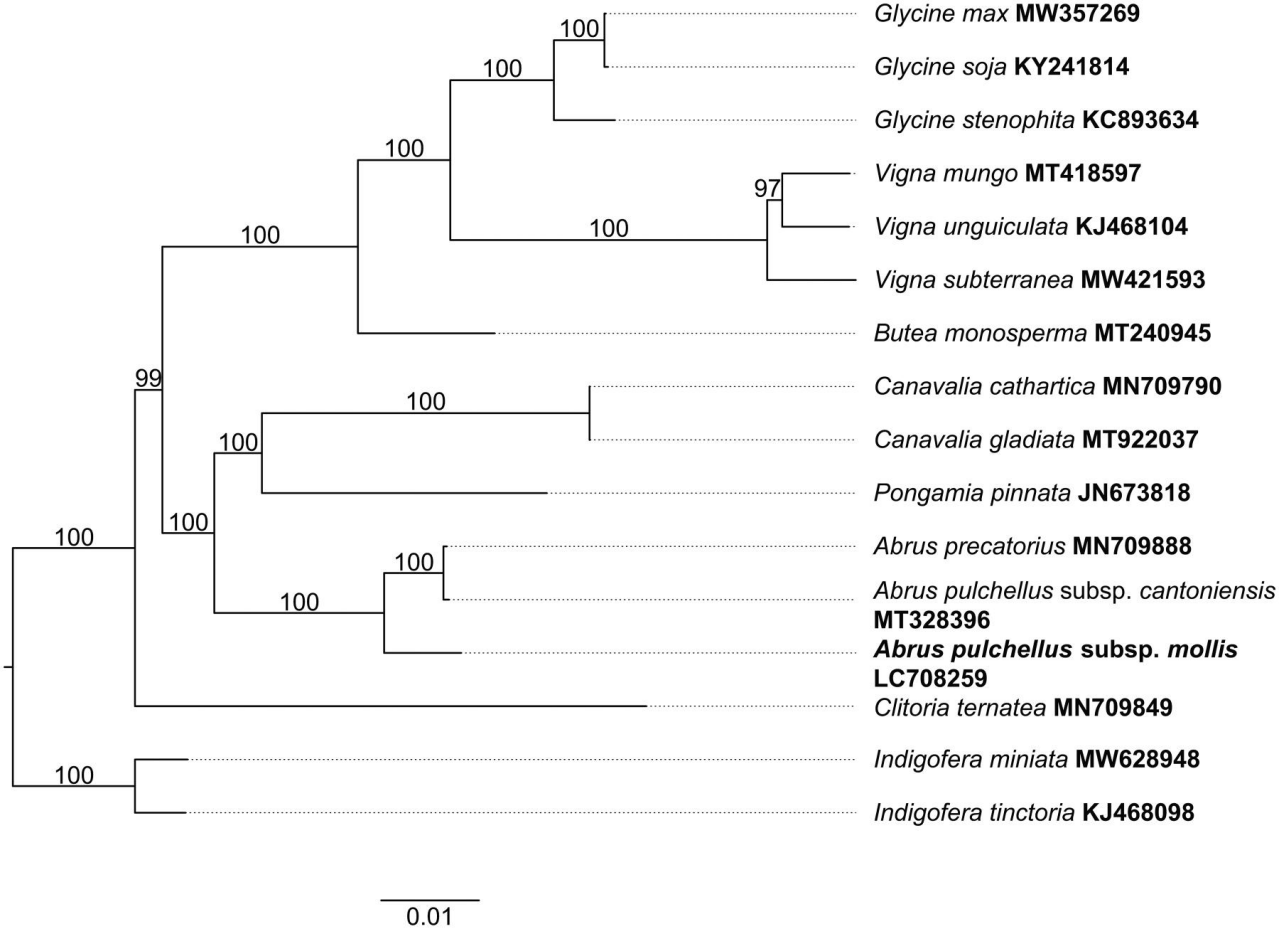
ML tree based on 16 complete plastid genomes of Millettioid/Phaseoloid clade of Leguminosae. The following plastomes are used: *Glycine max* (MW357269) (unpublished), *Glycine soja* (KY241814) (Asaf et al. 2017), *Glycine stenophita* (KC893634) (Sherman-Broyles et al. 2014), *Vigna mungo* (MT418597) (Nawae et al. 2020), *Vigna unguiculata* (KJ468104) (Schwarz et al. 2015), *Vigna subterranea* (MW421593) (unpublished), *Butea monosperma* (MT240945) (Wei et al. 2020), *Canavalia cathartica* (MN709790) (Zhang et al. 2020), *Canavalia gladiata* (MT922037) (Zhao 2021), *Pongamia pinnata* (JN673818) (Kazakoff et al. 2012), *Abrus precatorius* (MN709888) (Zhang et al. 2020), *Abrus pulchellus* subsp*. cantoniensis* (MT328396) (Xu et al. 2022), *Abrus pulchellus* subsp*. mollis* (LC708259) (this study), *Clitoria ternatea* (MN709849) (Zhang et al. 2020), *Indigofera miniate* (MW628948) (Lee et al. 2021), and *Indigofera tinctoria* (KJ468098) (Schwarz et al. 2015). Numbers along branches indicate the ML bootstrap values (1000 replicates). *Abrus pulchellus* subsp. *mollis* is presented in bold.

The aligned matrix, consisting of 76 conserved CDS derived from 16 complete plastid genomes (including the newly sequenced plastome presented in this study) was conducted using MAFFT v7.490 (Katoh and Standley 2013). This matrix was used for ML tree construction. Our analysis revealed a cohesive clustering of the three *Abrus* taxa, specifically *A. pulchellus* subsp. *mollis*, *A. pulchellus* subsp. *cantoniensis*, and *A. precatorius*, as depicted in Figure 3. Notably, *A. pulchellus* subsp. *cantoniensis* formed a sister group with *A. precatorius*, but not to the subspecies *A. pulchellus* subsp. *mollis* (Figure 3).

## Discussion and conclusions

The subspecies *A. pulchellus* subsp. *mollis* has garnered significant interest due to its profound medicinal properties in southern China. However, prior research has predominantly concentrated on its medicinal constituents, as noted by Hu et al. (2019). Morphologically, this subspecies bears resemblance to *A. pulchellus* subsp. *cantoniensis*, yet previous phylogenetic data were limited. For instance, only a scant number of molecular fragments from *A. pulchellus* subsp. *mollis* are accessible on NCBI (https://www.ncbi.nlm.nih.gov/nuccore/), encompassing a few plastid fragments such as *rbcL* and *matK*. Consequently, the precise phylogenetic relationships among the two subspecies of *A. pulchellus* and their closely related taxa within the same genus remain obscure.

With the advent of advanced sequencing technologies and the continual evolution of plastid genome assembly and analysis methodologies, the plastome has emerged as a pivotal instrument for current phylogenetic plant studies and the authentication of Chinese medicinal herbs, as highlighted by Chen et al. (2023) and Guo et al. (2023). This study marks the inaugural report on the structure and genetic composition of the plastome of *A. pulchellus* subsp. *mollis*, alongside a preliminary reconstruction of its phylogenetic ties with closely related taxa. The phylogenetic analysis of the plastid genomes reveals that *A. pulchellus* subsp. *cantoniensis* clusters in a sister group with *A. precatorius* and subsequently forms a clade with *A. pulchellus* subsp. *mollis*. These finding underscore substantial differences between the plastid genomes of the two subspecies, thereby establishing a groundwork for future research on the identification of medicinal herbs and germplasm resources pertaining to these subspecies.

## Supplementary Material

Supplementary Materials_June19.docx

## Data Availability

The plastid genome sequence of *A. pulchellus* subsp. *mollis* has been deposited in GenBank of NCBI with accession number LC708259 (https://www.ncbi.nlm.nih.gov/nuccore/LC708259). The associated BioProject, SRA, and BioSample accession numbers are PRJNA809489, SRP361020, and SAMN26179668, respectively.

## References

[CIT0001] Asaf S, Khan AL, Aaqil Khan M, Muhammad Imran Q, Kang SM, Al-Hosni K, Jeong EJ, Lee KE, Lee IJ. 2017. Comparative analysis of complete plastid genomes from wild soybean (*Glycine soja*) and nine other *Glycine* species. PLOS One. 12(8):e0182281. doi:10.1371/journal.pone.0182281. 28763486 PMC5538705

[CIT0002] Bao BJ, Gilbert MG. 2010. *Abrus*. In: Wu ZY, Raven PH, Hong DY, editors. Flora of China. Vol. 10. Beijing: Science Press; p. 194–195.

[CIT0003] Bolger AM, Lohse M, Usadel B. 2014. Trimmomatic: a flexible trimmer for Illumina sequence data. Bioinformatics. 30(15):2114–2120. doi:10.1093/bioinformatics/btu170. 24695404 PMC4103590

[CIT0004] Chen S, Yin X, Han J, Sun W, Yao H, Song J, Li X. 2023. DNA barcoding in herbal medicine: retrospective and prospective. J Pharm Anal. 13(5):431–441. doi:10.1016/j.jpha.2023.03.008. 37305789 PMC10257146

[CIT0005] Chinese Pharmacopoeia Commission. 2020. Pharmacopoeia of the People’s Republic of China. Beijing: China Medical Science Press.

[CIT0006] Doyle JJ, Doyle JL. 1987. A rapid DNA isolation procedure for small quantities of fresh leaf tissue. Phytochem Bull. 19:11–15.

[CIT0007] Guo C, Luo Y, Gao LM, Yi TS, Li HT, Yang JB, Li DZ. 2023. Phylogenomics and the flowering plant tree of life. J Integr Plant Biol. 65(2):299–323. doi:10.1111/jipb.13415. 36416284

[CIT0008] Hu XL, Niu YJ, Chen M, Feng JH, Shen W, Jiang ZZ, Zhang XQ, Ye WC, Xiong F, Wang H. 2019. Preventive effects of total flavonoid C-glycosides from *Abrus mollis* on nonalcoholic fatty liver disease through activating the PPARα signaling pathway. Planta Med. 85(8):678–688. doi:10.1055/a-0895-5838. 31026873

[CIT0009] Jin JJ, Yu WB, Yang JB, Song Y, DePamphilis CW, Yi TS, Li DZ. 2020. GetOrganelle: a fast and versatile toolkit for accurate de novo assembly of organelle genomes. Genome Biol. 21(1):241. doi:10.1186/s13059-020-02154-5. 32912315 PMC7488116

[CIT0010] Katoh K, Standley DM. 2013. MAFFT multiple sequence alignment software version 7: improvements in performance and usability. Mol Biol Evol. 30(4):772–780. doi:10.1093/molbev/mst010. 23329690 PMC3603318

[CIT0011] Kazakoff SH, Imelfort M, Edwards D, Koehorst J, Biswas B, Batley J, Scott PT, Gresshoff PM. 2012. Capturing the biofuel wellhead and powerhouse: the chloroplast and mitochondrial genomes of the leguminous feedstock tree *Pongamia pinnata*. PLOS One. 7(12):e51687. doi:10.1371/journal.pone.0051687. 23272141 PMC3522722

[CIT0012] Langmead B, Salzberg SL. 2012. Fast gapped-read alignment with Bowtie 2. Nat Methods. 9(4):357–359. doi:10.1038/nmeth.1923. 22388286 PMC3322381

[CIT0013] Laslett D, Canback B. 2004. ARAGORN, a program for the detection of transfer RNA and transfer-messenger RNA genes in nucleotide sequences. Nucleic Acids Res. 32:11–16. 14704338 10.1093/nar/gkh152 PMC373265

[CIT0014] Lee C, Choi IS, Cardoso D, de Lima HC, de Queiroz LP, Wojciechowski MF, Jansen RK, Ruhlman TA. 2021. The chicken or the egg? Plastome evolution and an independent loss of the inverted repeat in papilionoid legumes. Plant J. 107(3):861–875. doi:10.1111/tpj.15351. 34021942

[CIT0015] Liu SY, Ni Y, Li JL, Zhang XY, Yang HY, Chen HM, Liu C. 2023. CPGView: a package for visualizing detailed chloroplast genome structures. Mol Ecol Resour. 23(3):694–704. doi:10.1111/1755-0998.13729. 36587992

[CIT0016] Nawae W, Yundaeng C, Naktang C, Kongkachana W, Yoocha T, Sonthirod C, Narong N, Somta P, Laosatit K, Tangphatsornruang S, et al. 2020. The genome and transcriptome analysis of the *Vigna mungo* chloroplast. Plants. 9(9):1247. doi:10.3390/plants9091247. 32967378 PMC7570002

[CIT0017] Schwarz EN, Ruhlman TA, Sabir JS, Hajrah NH, Alharbi NS, Al‐Malki AL, Bailey CD, Jansen RK. 2015. Plastid genome sequences of legumes reveal parallel inversions and multiple losses of *rps16* in papilionoids. J Syst Evol. 53(5):458–468. doi:10.1111/jse.12179.

[CIT0018] Shen W, Hu X, Niu Y, Lu Y, Wang B, Wang H. 2021. Bioaccessibility and absorption of flavonoid C-glycosides from *Abrus mollis* using simulated digestion, Caco-2 Cell, and in situ single-pass perfusion models. Planta Med. 87(7):570–580. doi:10.1055/a-1363-2088. 33545720

[CIT0019] Sherman-Broyles S, Bombarely A, Grimwood J, Schmutz J, Doyle J. 2014. Complete plastome sequences from *Glycine syndetika* and six additional perennial wild relatives of soybean. G3. 4(10):2023–2033. doi:10.1534/g3.114.012690. 25155272 PMC4199708

[CIT0020] Stamatakis A. 2014. RAxML version 8: a tool for phylogenetic analysis and post-analysis of large phylogenies. Bioinformatics. 30(9):1312–1313. doi:10.1093/bioinformatics/btu033. 24451623 PMC3998144

[CIT0021] Tillich M, Lehwark P, Pellizzer T, Ulbricht-Jones ES, Fischer A, Bock R, Greiner S. 2017. GeSeq – versatile and accurate annotation of organelle genomes. Nucleic Acids Res. 45(W1):W6–W11. doi:10.1093/nar/gkx391. 28486635 PMC5570176

[CIT0022] Wei D, Ding X, Zhu H, Zhang Z, Yang H, Zhou R, Dai S, Zhang G. 2020. The complete chloroplast genome sequence of *Butea monosperma* (Fabaceae). Mitochondrial DNA B Resour. 5(3):3255–3256. doi:10.1080/23802359.2020.1811173. 33458130 PMC7781871

[CIT0023] Wick RR, Schultz MB, Zobel J, Holt KE. 2015. Bandage: interactive visualization of de novo genome assemblies. Bioinformatics. 31(20):3350–3352. doi:10.1093/bioinformatics/btv383. 26099265 PMC4595904

[CIT0024] Xu S, Sun M, Mei Y, Gu Y, Huang D, Wang J. 2022. The complete chloroplast genome sequence of the medicinal plant *Abrus pulchellus* subsp. *cantoniensis*: genome structure, comparative and phylogenetic relationship analysis. J Plant Res. 135(3):443–452. doi:10.1007/s10265-022-01385-w. 35338406

[CIT0025] Zhang R, Wang Y-H, Jin J-J, Stull GW, Bruneau A, Cardoso D, De Queiroz LP, Moore MJ, Zhang S-D, Chen S-Y, et al. 2020. Exploration of plastid phylogenomic conflict yields new insights into the deep relationships of Leguminosae. Syst Biol. 69(4):613–622. doi:10.1093/sysbio/syaa013. 32065640 PMC7302050

[CIT0026] Zhao J. 2021. Characterization of the complete chloroplast genome of *Canavalia gladiata*. Mitochondrial DNA B Resour. 6(1):252–253. doi:10.1080/23802359.2020.1861566. 33553637 PMC7850338

