# Supplementary material for: The complete plastid genome of Abrus pulchellus subsp. mollis (Leguminosae): a medicinal plant in Southern China
Source: Mitochondrial DNA B Resour. 2024 Jul 29;9(7):943–7. doi: 10.1080/23802359.2024.2383684 (PMC11288203; doi:10.1080/23802359.2024.2383684)
Supplement: Supplementary Materials_June19.docx [file TMDN_A_2383684_SM4538.docx]

**Supplementary Materials**

**Figure S1.** Mapping of the quality of the plastome assembly of *Abrus pulchellus* subsp. *mollis* (LC708259). Coverage scale = log(coverage+1) + log(maximumcoverage+1).

**Figure S2.** Schematic map of cis-splicing genes in the plastome of *Abrus pulchellus* subsp. *mollis* generated using CPGview. The exons are shown in black and the introns are shown in white. The arrows indicate the sense direction of the genes.

**Figure S3**. Schematic map of trans-splicing *rps12* in the plastome of *Abrus pulchellus* subsp. *mollis* generated using CPGview*.* The arrows indicate the sense direction of the genes.
